# Profile of Modifiable and Non-Modifiable Risk Factors in Stroke in a Rural Based Tertiary Care Hospital – A Case Control Study

**DOI:** 10.5539/gjhs.v4n3p158

**Published:** 2012-05-01

**Authors:** Aniruddha Deoke, Shilpa Deoke, Ajeet Saoji, Shilpa Hajare

**Affiliations:** 1 Department of Community Medicine, NKP Salve Institute of Medical Sciences and Research Center, Nagpur, India; 2 Department of Medicine, NKP Salve Institute of Medical Sciences and Research Center, Nagpur, India

**Keywords:** stroke, modifiable, non-modifiable, risk factors

## Abstract

**Background:**

Stroke, a major public health problem in India and worldwide, is associated with many risk factors. The modification of risk factors, an important public health strategy, has been shown to reduce the risk of stroke. Hence the present study was carried out to document the risk factor profile of stroke.

**Methods:**

It was a case-control study. Patients with stroke admitted in a tertiary care centre in central India and age and sex matched controls were included. Detail history and clinical examination was done in all cases and controls. The risk factors studied were education, socioeconomic status (according to Kuppuswamy’s classification), level of physical activity, alcohol intake, and smoking, tobacco chewing, family history of stroke and history of systemic hypertension, transient ischemic attack or ischemic heart disease. Anthropometric (weight, height, body mass index and waist circumference) measurements were done in all patients. Electrocardiogram was done in cases as well as controls and abnormalities noted.

**Statistical Analysis:**

The data was analyzed using Epi info version 3.4.1 software. Chi-square test was used as test of significance and p value less than 0.05 was considered as significant.

**Results:**

On comparing the cases with controls, sedentary life-style (p=0.02), history of transient ischemic attack (p=0.002), coronary artery disease (p=0.014), family history of stroke (p=0.001), systemic hypertension (p<0.001) and ECG abnormalities (p=0.04) were significant risk factors whereas low socio-economic status (p=0.40), smoking (p=0.12), tobacco chewing (p=0.35), alcohol consumption (p=0.22), obesity [both central and generalized as assessed by waist circumference (p=0.33) and BMI respectively (p=0.43)] and Diabetes mellitus (p=0.07) were not found to be statistically significant risk factors. The most significant risk factor was systemic hypertension (OR= 15.92, 95% CI, 1.78-6.85) followed by coronary artery disease (OR=3.86, 95% CI, 1.13-14.50), abnormal ECG (OR=2.49, 95% CI, 0.97-6.96) and sedentary life-style (OR=2.41, 95% CI, 1.07-5.49).

**Conclusions:**

In the present hospital based case control study in patients with stroke, sedentary life-style, history of transient ischemic attack, family history of stroke, coronary artery disease, systemic hypertension and abnormal ECG were significant risk factors. This could be helpful in early identification of subjects at risk for stroke and formulating public health strategy, if proven by larger population based studies.

## 1. Introduction

Stroke or cerebrovascular episode is a major public health problem globally and the third leading cause of death worldwide after coronary heart disease and cancer (Murray et al., 1996). According to WHO estimates, stroke accounted for 5.7 million deaths worldwide. In India, prevalence rate of stroke is 1.54 with a death rate of 0.6 per 1000 population. Furthermore, a significant proportion of survivors have long term disabilities due to neurological sequelae. Thus it has enormous socioeconomic impact on the patients, their families and health services.

Due to its huge burden on public health and its considerable socioeconomic impact, prevention of stroke and its associated risk factors has, in recent times, become an important public health priority. The various risk factors for stroke are increasing age, male sex, smoking, hypertension, Diabetes mellitus, dyslipidaemia, ischemic heart disease, obesity; lack of physical activity, alcohol consumption and other rare causes (Martin et al., 2010; Boysen et al., 1988). Modification of risk factors has been shown to reduce the risk of stroke (Futterman et al., 1999).

The present study was carried out to study the type of stroke and the various modifiable and non-modifiable risk factors for stroke.

## 2. Methods

The study was initiated after consent from the Institutional Ethics Committee. The study was conducted over a one year period at a tertiary referral centre in central India. It was a hospital based case control study.

### 2.1 Cases

Patients with stroke admitted in wards and ICU in a tertiary care hospital were included. Daily rounds in the morning hours were taken and patients with completed stroke were studied. Diagnosis was made on clinical and/or neuro-imaging (CT/MRI) findings. According to World Health Organization criteria, stroke was defined as rapidly developing clinical symptoms and/or signs of focal or global cerebral dysfunction with symptoms lasting more than 24 hours or with death, of apparent vascular origin. Patients who died within 24 hours of hospitalization were excluded. After taking informed consent, detail history and clinical examination was carried out. In case of aphasic or comatose patients, the history was given by either the spouse or a first degree relative that lived in the same house or was aware of the patient’s past and present treatment. Subjects or relatives not willing to participate or in whom history could not be elicited due to non-availability of a first degree relative or spouse were also excluded.

### 2.2 Controls

Age (+/-5 years) and sex matched individuals without history of stroke or transient ischemic attack admitted for other illness were taken as controls.

Assessment of risk factors: A pre-validated questionnaire detailing the education, socioeconomic status (according to Kappu Swamy’s classification), level of physical activity, alcohol intake, smoking, tobacco chewing, family history of stroke and history of systemic hypertension, transient ischemic attack or ischemic heart disease was administered in all patients. Anthropometric (weight, height, body mass index and waist circumference) measurements were done in all patients. Waist circumference was measured in standing or supine position (for comatose patients or patients with physical disability). Waist circumference more than 80cm in females and more than 90 cm in males was considered abnormal. For BMI, a cut-off of 25 kg/m2 was considered. According to the level of education, patients were divided into 2 groups; illiterate or primary schooling were classified as one group and the other group included patients with higher education (upto post graduation). Socioeconomic status of cases and controls was decided by the Kappu Swamy’s classification (Kappu Swamy, 1976). Sedentary life-style was defined as less than 25 minutes of physical activity per day (Antonio Cabrera de León et al., 2007). Subjects were classified as smokers if they were present or ex-smokers. Similarly subjects were classified as alcoholic if they were present or ex-alcoholic. Subjects were classified as hypertensive if the blood pressure was more than 140/90 mm of Hg or there was a self-reported history of hypertension or history of receiving anti-hypertensive medications. History of Diabetes mellitus was confirmed either by a self-reported history or history of receiving anti-diabetic medications or fasting plasma glucose more than 126 mg%. Subjects were said to have coronary artery disease (CAD) if there was present or past history of angina or the ECG showed ischemic changes (Q wave or ST segment - T wave changes). Transient ischemic attack was defined as reversible, focal neurological deficit recovering within 24 hours. Family history of stroke in the first degree relatives was noted. Electrocardiogram was done in cases as well as controls and abnormalities noted.

### 2.3 Statistical Analysis

The data was analyzed using Epi info version 3.4.1 software. Chi-square test was used as test of significance and p value less than 0.05 was considered as significant. Whenever the cell number was less than 5, Fischer exact test was used.

## 3. Results

110 patients of completed stroke were admitted during the study period, of which 2 expired within 24 hours of hospitalization and in 7 patients questionnaire could not be administered due to non-availability of a spouse or a first degree relative. Finally 101 cases (65 male, 36 female) and 100 age and sex matched controls were included in the study. Mean age of cases and controls was 59.30+-12.44years and 60+-12.16 years respectively. The male:female ratio was 1.81:1 and 1.78:1 in cases and controls respectively. Most (n= 54, 53.47%) were elderly (>60 years) (Table 1). There were 86 ischemic (54 male and 32 female) and 15 cases of hemorrhagic stroke (11 male and 4 female) (Table 2). Focal motor weakness was the commonest clinical presentation (n=81, 80.12%) followed by speech abnormality (dysphasias) (n=36, 35.64%), altered sensorium and seizures (6 each, 5.94%) (Table 3).

**Table 1:** Age and sex distribution of cases and controls

Age (years)	Cases		Controls	

	Male (n=65)	Female (n=36)	Male (n= 64)	Female (n=36)
<39	4	3	3	3
40-59	27	13	27	13
>60	34	20	34	20
Total	65	36	64	36

**Table 2:** Distribution of cases according to the type of stroke

Type of stroke	Male (n=65)	Female (n= 36)	Total (n= 101)
Ischemic	54	32	86
Hemorrhagic	11	4	15
Total	65	36	101

**Table 3:** Clinical presentations of patients with stroke

Presentation	Number of cases (n=101)	Percentage (%)
Focal motor weakness	81	80.12
Speech abnormalities (Dysphasias, Dysarthria)	36	35.64
Altered sensorium	6	5.94
Seizures	6	5.94
Others	5	4.95

*Multiple responses were allowed

The profile of risk factors in cases and controls is described in table 4. On comparing the cases with controls, low socio-economic status (p=0.40), smoking (p=0.12), tobacco chewing (p=0.35), alcohol consumption (p=0.22), obesity [both central and generalized as assessed by waist circumference (p=0.33) and BMI respectively (p=0.43)] and Diabetes mellitus (p=0.07) were not found to be statistically significant. Sedentary life-style (p=0.02), history of transient ischemic attack (p=0.002), family history of stroke (p=0.001), coronary artery disease (p=0.014), systemic hypertension (p<0.001) and abnormal ECG (p=0.04) were significant risk factors.

**Table 4:** Profile of risk factors in cases and controls

Risk factor	Cases (n=101)	Controls (n=100)	‘p’ value	Odds Ratio
Mean age(years)	59.30±12.44	60±12.16		
Education (Upto Primary)	38	34	0.59	
Low socioeconomic status (Kuppuswamy I+II)	25	30	0.40	
Smoking	32	22	0.12	
Tobacco chewing	29	23	0.35	
Alcohol	20	27	0.22	
*Sedentary life-style	25	12	0.02	2.41 (CI: 1.07-5.49)
BMI >25kg/m2	16	12	0.43	
Increased Waist circumference	17	12	0.33	
*Hypertension	47	20	<0.001	15.92(CI:1.78-6.85)
Diabetes mellitus	23	13	0.07	
*Coronary artery disease	14	4	0.014	3.86(CI:1.13-14.50)
*History of TIA#	9	0	0.002	
*Family history of stroke#	10	0	0.001	
*Abnormal ECG	18	8	0.04	2.49(CI:0.97-6.96)

* statistically significant variable

#Fisher exact test

## 4. Discussion

Age and male sex are important non-modifiable risk factors for stroke. These observations are affirmed in the present study as most patients were above 60 years of age (n=54, 53.47%) and male preponderance was observed in ischemic as well as hemorrhagic stroke (male: female ratio 1.81:1 3.75:1 respectively).

In the present study, significantly more cases had sedentary life-style (p=0.02, OR-2.41, 95%CI 1.07-5.49). Protective effect of physical activity on stroke incidence in men and women has been observed in previous studies also (Abbott et al., 1994; Kiely et al., 1994). Physical activity conferred protection in men (adjusted relative risk was 0.41) but not in women in the Framingham study. Further, heavy physical activity was not found to be more beneficial than moderate activity (27% and 20% lower risk for stroke occurrence or mortality, respectively, than low activity). However, stroke rates were lower in moderately and highly active individuals than in individuals with low activity (Lee et al., 2003). The beneficial effects of physical activity are observed due to reduction in blood pressure, weight, and pulse rate; favorable modification of the lipid profile (increase in HDL cholesterol and decrease in LDL cholesterol); reduction in platelet aggregability and improvement in glycaemic control by improving insulin sensitivity.

In the present study, 9 (8.91%) cases had history of TIA whereas none of the controls had history of TIA. Thus history of TIA was a significant risk factor (p=0.002). Similar observations were made by Rodgers H et al who observed a hazard ratio of 1.187 (95% CI, 1.27 – 2.76) for TIA in the elderly people of North-east England with first ever stroke (Rodgers et al., 2004).

Presence of CAD was a significant risk factor in cases in the present study (p=0.014, OR=3.86, 95% CI, 1.13 -14.50). Similarly, in a large population based study CAD was a significant risk factor for stroke (Boysen et al., 1988). In their study of classic risk factors, Rodgers H et al noted a hazard ratio of 1.55(95%, CI, 1.19 – 2.03) for CAD (Rodgers et al., 2004). Many risk factors (Hypertension, Diabetes mellitus, dyslipidaemia, lack of exercise) are frequent in both, CAD and stroke. Further, atherosclerosis, an invariable pathogenetic process in CAD is also a common pathophysiologic mechanism in stroke. Thus common risk factors and common pathogenesis are probably responsible for the increased risk of stroke in patients with CAD.

While family history of stroke was present in 10 cases, none of the controls had a positive history (p=0.02). Familial clustering of stroke has also been demonstrated previously. When familial risk of stroke was studied, a strong association of stroke between siblings was observed and genetic factors were suggested as predictors of stroke (Sundquinst et al., 2006). Similarly, Tentschert et al. demonstrated correlation between maternal history of stroke and prevalence of hypertension and left ventricular hypertrophy in female patients with ischemic stroke (Tentschert et al., 2003). In the Framingham Study, paternal as well as maternal history of stroke was found to be associated with an increased risk (Kiely et al., 1993). However, Boysen et al could not demonstrate a significant correlation with positive family history of stroke (Boysen et al., 1988). Genetic and/or environmental factors or common lifestyle risks (Kiely et al., 1993) are probably responsible for familial clustering of stroke. Furthermore, many classical risk factors like Hypertension, Diabetes mellitus and dyslipidaemia also have a common genetic basis and thus can be seen in families.

Systemic hypertension was observed to be the most significant risk factor for stroke in the present study (OR=15.92. 95% CI, 1.78 – 6.85). In the INTERSTROKE study, history of hypertension was a significant risk factor for all strokes (OR 2.64, 99% CI 2.26-3.08; PAR 34.6%, 99%CI 18.8-36.6) (Martin et al., 2010). Similarly, the estimated 10 year risk of stroke in the elderly population of Spain was 19.6%; the risk was more in hypertensive patients (23.7%; standard deviation, 18.5) than in patients with high blood pressure without known hypertension (12.4%; standard deviation, 9.2), or in normotensive subjects (5.3%; standard deviation.0.2; p<0.001) (Redon et al., 2007). Likewise, for each 10 mm Hg rise in systolic and diastolic blood pressure higher relative risks of total stroke were noted in Asian group than in whites (1.2 to1.3 and 1.2 to 1.5, respectively). The population attributable risk of hypertension to stroke was higher in Asians (i.e., 31% for ischemic and 42% for hemorrhagic stroke) than in whites (25% and 34%, respectively) (Zhang et al., 2004). Hazard ratio was 1.15 (95% CI, 1.06 to 1.24) for every 10 mm Hg rise in systolic blood pressure in the study by Rodgers H et al (Rodgers, et al., 2004).

Significantly more cases had ECG abnormalities as compared to controls (p=0.04, OR=2.49, 95% CI, 0.97-6.96). While the most common abnormality was left ventricular hypertrophy (LVH) (n=12, 11.88%) (voltage criteria of Sokolow (Sokolow et al., 1949), ST-T changes suggestive of Coronary artery disease were present in 10 (9.90%) and 2 (1.98%) patients had atrial fibrillation. Redon J et al analyzed 7343 elderly subjects and observed that Electrocardiographic left ventricular hypertrophy was present in 12.9% of the subjects and atrial fibrillation was present in 8.4% (Redon et al., 2007). However, these abnormalities may be a consequence of systemic hypertension (LVH) or co-existent CAD rather than a cause of stroke. Furthermore, ECG changes might also be observed due to stroke per se.

Small sample size of the present study is undoubtedly a limitation of the present study. Hence to corroborate its findings, larger, prospective, population based studies are further required.

## 5. Conclusions

To conclude, in the present hospital based case control study, sedentary life-style (p=0.02), history of transient ischemic attack (p=0.002), coronary artery disease (p=0.014), family history of stroke (p=0.001), systemic hypertension (p<0.001) and ECG abnormalities (p=0.04) were significant risk factors in patients with stroke, whereas low socio-economic status (p=0.40), smoking (p=0.12), tobacco chewing (p=0.35), alcohol consumption (p=0.22), obesity [both central and generalized as assessed by waist circumference (p=0.33) and BMI respectively (p=0.43)] and Diabetes mellitus (p=0.07) were not found to be statistically significant risk factors.

Most of the significant risk factors in the present study are potentially modifiable and increased awarenes about the primordial prevention of these risk factors may be beneficial to the population at risk. Due to the sheer magnitude, devastating consequences and residual sequelae of stroke, early intervention in the form of patient education, lifestyle modification and non-pharmacological as well as pharmacological interventions for the modifiable risk factors should form an integral aspect of patient care.
